# Changes in Choroidal Thickness follow the RNFL Changes in Leber’s Hereditary Optic Neuropathy

**DOI:** 10.1038/srep37332

**Published:** 2016-11-17

**Authors:** Enrico Borrelli, Giacinto Triolo, Maria Lucia Cascavilla, Chiara La Morgia, Giovanni Rizzo, Giacomo Savini, Nicole Balducci, Paolo Nucci, Rosa Giglio, Fatemeh Darvizeh, Vincenzo Parisi, Francesco Bandello, Alfredo A. Sadun, Valerio Carelli, Piero Barboni

**Affiliations:** 1 Department of Ophthalmology, San Raffaele Scientific Institute, Milan, Italy; 2 IRCCS, Istituto delle Scienze Neurologiche di Bologna, Bellaria Hospital, Bologna, Italy; 3 Neurology unit of the Department of Biomedical and Neuromotor Sciences (DIBINEM), University of Bologna, Bologna, Italy; 4 G.B. Bietti Foundation, Rome, Italy; 5 Studio Oculistico d’Azeglio, Bologna, Italy; 6 San Giuseppe Hospital, University Eye Clinic, Milan, Italy; 7 Doheny Eye Institute, University of California at Los Angeles (UCLA), Los Angeles, CA, USA

## Abstract

Leber’s hereditary optic neuropathy (LHON) is typically characterized by vascular alterations in the acute phase. The aim of this study was to evaluate choroidal changes occurring in asymptomatic, acute and chronic stages of LHON. We enrolled 49 patients with LHON, 19 with Dominant Optic Atrophy (DOA) and 22 healthy controls. Spectral Domain-Optical Coherence Tomography (SD-OCT) scans of macular and peripapillary regions were performed in all subjects, to evaluate macular and peripapillary choroidal thickness, and retinal nerve fiber layer (RNFL) thicknes. Macular and peripapillary choroidal thicknesses were significantly increased in the acute LHON stage. On the contrary, macular choroidal thickness was significantly reduced in the chronic stage. Furthermore, peripapillary choroidal thickness was decreased in chronic LHON and in DOA. Both RNFL and choroid had the same trend (increased thickness, followed by thinning), but RNFL changes preceded those affecting the choroid. In conclusion, our study quantitatively demonstrated the involvement of the choroid in LHON pathology. The increase in choroidal thickness is a feature of the LHON acute stage, which follows the thickening of RNFL. Conversely, thinning of the choroid is the common outcome in chronic LHON and in DOA.

Optic nerve damage has been recognized as a frequent feature of mitochondrial diseases. Leber’s Hereditary Optic Neuropathy (LHON) and Dominant Optic Atrophy (DOA) are both caused by mitochondrial dysfunction and are characterized by tissue selectivity, usually limited to damaging the retinal ganglion cells (RGCs) and their axons in the optic nerve^1^,^2^,^3^,^4^.

LHON is a maternally inherited genetic disorder caused by mitochondrial DNA (mtDNA) point mutations affecting different subunits of complex I and leading to dysfunction in the mitochondrial respiratory chain. The three most frequent pathogenic mutations are at nucleotide positions 11778/ND4, 3460/ND1, and 14484/ND6. Clinically, LHON is characterized by simultaneous or sequential acute loss of visual acuity with bilateral central scotomas that occur most frequently in young men^2^,^4^. The smaller-caliber fibers of the papillomacular bundle are selectively lost in early stages of the pathologic process^5^,^6^,^7^. Fiber loss eventually progresses, as a wave, to ultimately involve the entire optic nerve and manifest as optic atrophy^8^.

Asymptomatic carriers of the mtDNA LHON mutations and especially carriers about to convert before the acute phase of the disease often show classical ophthalmoscopic changes: circumpapillary telangiectatic microangiopathy, small vessel tortuosity, and swelling of the RNFL inferior-temporally^8^,^9^,^10^. When the disease becomes symptomatic and the patient experiences loss of central vision, there may be a concomitant increase in microangiopathy accompanied by swelling of the superior and inferior fiber arcades and rapid loss of the papillomacular bundle^8^,^11^. Over a period of weeks to months, the microangiopathy and nerve fiber swelling decrease and unmask extensive optic disc atrophy. By 6 months after onset, optic atrophy is usually pronounced and visual loss stabilizes. The pathophysiologic mechanisms of disease at conversion remain unclear with several hypotheses having been proposed that usually involve increased reactive oxygen species (ROS) accumulation, reduced ATP availability, and stasis of axoplasmic flow due to impaired axonal transport. Less understood however, are the vascular changes as reflected by the telangiectasias. Thus, the question arises, is there a primary micro-vascular disorder in LHON?

DOA, at difference with LHON, is a neurodegenerative disorder associated with dominant mutations in the nuclear gene *OPA1*, which encodes a dynamin-related GTPase targeted to mitochondria and involved in the fusion of mitochondrial inner membrains^4^,^12^,^13^,^14^. Clinically, DOA is characterized by a slowly progressive bilateral visual loss, starting in childhood and ultimately leading to various degrees of optic atrophy^15^,^16^. Both the visual loss and the optic atrophy are not usually as severe in DOA as in LHON^1^,^2^.

Advances in ocular imaging, with the introduction of the spectral domain optical coherence tomography (SD-OCT) have enabled clinicians to visualize in detail retinal and choroidal structures, the latter using the enhanced depth imaging (EDI) technique^17^. Recently, SD-OCT has been instrumental in the qualitative and quantitative assessment of funduscopic changes over the natural history of LHON^8^,^18^,^19^. Nevertheless, OCT studies have been focused on measuring RNFL and RGC thickness, whereas the vascular changes remained neglected and poorly evaluated. A few studies have shown vascular changes occurring in inflammatory papillitis^20^,^21^, nevertheless none tested the choroidal thickness in mitochondrial optic neuropathies. The aim of this study is to fill this gap by investigating choroidal thickness in asymptomatic, acute and chronic stages of LHON. Moreover, we compared choroidal thickness in two mitochondrial diseases causing optic atrophy, LHON in its chronic stage and DOA.

## Methods

### Subjects

Forty-nine consecutive LHON patients (98 eyes) were recruited for the study, all at the University Eye Clinic of San Raffaele Hospital between September 2012 and June 2015. All participants gave their informed consent according to the Declaration of Helsinki and the study was approved by the internal review board at the University Eye Clinic of San Raffaele Hospital, Milan. Moreover, all the subjects were tested in accordance with relevant guidelines and regulations.

All patients included in the present study had a molecularly confirmed diagnosis of LHON with an mtDNA primary mutation. Both eyes of all patients were considered for the analysis. We divided LHON eyes into 3 groups: affected eyes during the acute phase (A-LHON), when the disease onset was less then 6 months before; affected eyes during the chronic phase (C-LHON), when the disease onset was more than 1 year and asymptomatic mutation carriers (LHON carriers). Two patients were classified as ‘dynamic’ in the study groups because they were experiencing the dynamic anatomical changes characterizing this stage of the disease (between 6 months and 1 year after onset).

Moreover, nineteen consecutive patients (38 eyes) affected by DOA were enrolled for the present study. All these patients had a diagnosis molecularly proved with an *OPA1* pathogenic mutation.

All subjects had an extensive ophthalmologic examination, including best-corrected visual acuity measurement, slit lamp biomicroscopy, intraocular pressure measurement, indirect ophthalmoscopy, and optic nerve head photography. Exclusion criteria were the presence of any retinal pathology and/or optic nerve disease other than either LHON or DOA, spherical and/or cylindrical refractive errors higher than 3 and 2 diopters respectively, systemic conditions that may affect the vascular system (e.g. autoimmune condition, diabetes, and uncontrolled systemic hypertension).

Controls (22 subjects, 44 eyes) were healthy subjects analyzed during a routine ophthalmological examination. Inclusion criteria were: best corrected visual acuity better than 20/25, refractive errors lower than 3 diopters of sphere and 2 diopters of cylinder, normal intraocular pressure (<18 mm Hg), normal appearance of the optic disc, normal visual field (the latter was examined with a SITA 24-2 standard test in all subjects using the Humphrey VF analyzer, HFA II 750-4.1 2005, Carl Zeiss Meditec Inc, USA), no significant ocular disease found by routine ophthalmological examination, no history of glaucoma in the family and/or systemic diseases with possible ocular involvement, such as diabetes. Controls were matched with patients for age and refractive error because these factors have been shown to influence OCT measurements^22^,^23^. Moreover, because it has been shown that cigarette smoking leading to a significant increase in choroidal thickness up to 1 h after^24^, all the subjects had not smoked the hour before the examination. Finally, in order to avoid circadian choroidal changes that could influence our results^25^, all the tests were done during the morning.

### Instrumentation and procedures

#### Enhanced depth imaging-optical coherence tomography

**Figure 1:**
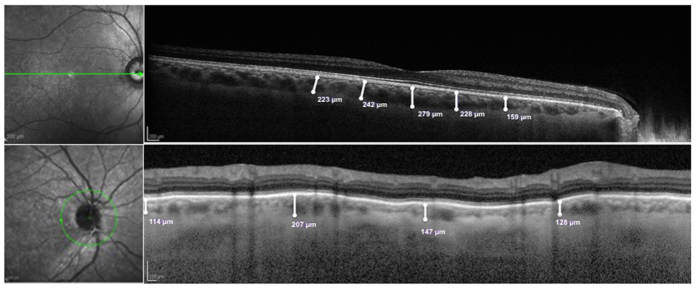
(Top Figure) Representative horizontal 9-mm high-quality line scan through the fovea, from a healthy subject (control group). The choroid was measured from the outer portion of the hyperreflective line corresponding to the retinal pigment epithelium to the inner surface of the sclera. These measurements were made of the sub-foveal choroid and at 750 μm intervals from the fovea to 1.5 mm nasal, 1.5 mm temporal, 1.5 mm superior, and 1.5 mm inferior from the center of the fovea. (Bottom Figure) Representative 360°, 3.4 mm diameter circle scan centered on the optic disc, from a healthy subject (control group). The choroid was measured from the outer portion of the hyperreflective line corresponding to the retinal pigment epithelium to the inner surface of the sclera. These measurements were made of four points: each point is far from the previous and the consecutive 90°.

SD-OCT scans of macular and peripapillary regions were performed using the Heidelberg Spectralis (version 1.7.0.0, Heidelberg Engineering, Heidelberg, Germany). The images were obtained by using enhanced depth imaging, which places the focus more posterior than would normally be done during standard retinal SD-OCT imaging, in order to improve the choroidal resolution^17^. Two 9-mm high-quality line scans through the fovea (one horizontal and one vertical) were obtained for each eye; the peripapillary region was scanned using a 360°, 3.4 mm diameter circle scan that was centered on the optic disc. The images were shown and measured with the Heidelberg Eye Explorer software. The choroid was measured in a masked fashion by a trained investigator (E.B.), after pupil dilation and in similar lighting and in the morning, from the outer portion of the hyper-reflective line corresponding to the retinal pigment epithelium (RPE) to the inner surface of the sclera. Macular measurements of the choroid thickness were made in the sub-foveal (SF) location and at 750 *μ*m intervals from the fovea up to 1.5 mm nasal, 1.5 mm temporal, 1.5 mm superior, and 1.5 mm inferior from the center of the fovea [Fig. 1]. The peripapillary choroidal thickness was measured at four points, each point being at 90° from the previous and consecutively, in order to investigate the temporal, superior, nasal and inferior peripapillary choroid thickness respectively [Fig. 1]. Furthermore, peripapillary RNFL thickness measurements were obtained by the same circle scan.

### Statistical analysis

Statistical calculations were performed using Statistical Package for Social Sciences (version 20.0. SPSS Inc. Chicago. IL. USA). To detect departures from normality distribution, the Kolmogorov-Smirnov test was performed for all variables. All quantitative variables were presented as mean and standard deviation (SD) in the results and in the tables. Parameters were compared by one-way analysis of covariance (ANCOVA), introducing sex and age as covariates, followed by Bonferroni post hoc test for pair wise comparisons. Pearson’s correlation was carried out among disease duration, best-corrected visual acuity, choroidal thickness and RNFL thickness measurements. In the correlation analysis, the A-LHON and C-LHON patients were grouped. Moreover, a regression analysis was focused on patients in the firsts 12 months from onset, including RNFL (average and temporal) and choroidal (average and temporal nerve and average and nasal macular) measurements.

The chosen level of statistical significance was p < 0.05.

## Results

### Demographic data of the investigated samples

Table 1 shows the demographic data of patients and healthy controls. Two eyes of 2 C-LHON patients had to be excluded from the analysis due to the poor quality of the scans.

### Choroidal and RNFL analyses

Table 2 separately compares A-LHON, LHON carriers and healthy subjects. Both macular and nerve choroidal thicknesses were significantly increased in A-LHON patients as compared with control group, except in nasal and inferior nerve measurements. The LHON carrier group showed an increased choroidal thickness that reached statistical significance only in the average and in the inferior nerve choroidal thicknesses, as compared with the control group. Moreover, A-LHON patients compared with carriers demonstrated a significant thickening of macular choroidal measurements (excluding the 1500 μm inferior one). Peri-optic nerve choroidal thickness measurements were increased, reaching significance only in the temporal sector, differently from comparable thickness in the inferior sectors.

Table 3 separately compares C-LHON, DOA and healthy subjects. Macular choroidal thickness was significantly reduced in C-LHON patients (in which thinning failed to reach statistical significance only in the 1500 μm nasal and in the 1500 μm inferior measurements) as compared with control group. Reduction in peri-optic nerve choroidal thickness between C-LHON patients and healthy subjects failed to reach statistical significance, except for the inferior measurement that had comparable thickness. DOA patients had a significantly thinner macular choroid in all measurements as compared with controls and a reduction in peri-optic nerve choroidal thickness measurements failed to reach statistical significance. Furthermore, no difference was found in choroidal thickness between C-LHON and DOA [Table 3].

RNFL thickness comparisons among A-LHON, LHON carriers, C-LHON, DOA and healthy subjects are shown in Tables 4 and 5. In A-LHON patients RNFL thickness was significantly higher in average, superior and inferior measurements compared to both controls and carrier groups, whereas temporal thickness was similar when compared with controls and reduced when compared with carriers [Table 4]. In carriers, RNFL thickness was higher in average, temporal and inferior measurements in comparison to controls.

RNFL thickness was significantly thinner in both C-LHON and DOA groups, as compared with controls. Finally, RNFL was significantly thinner in the C-LHON group as compared with DOA (except for the temporal measurement) [Table 5].

**Figure 2:**
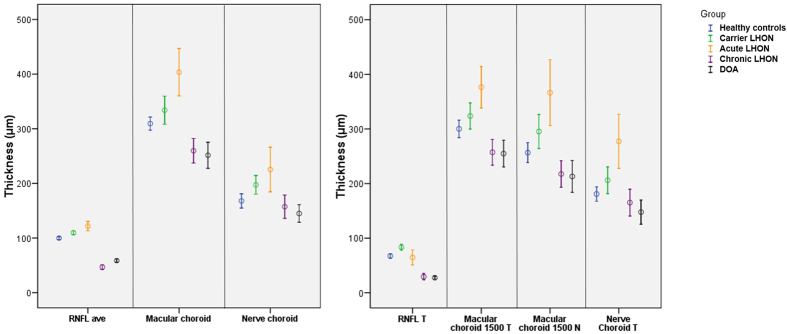
Boxplot of average retinal nerve fiber layer (RNFL), macular and nerve choroid (left panel) thickness, and of temporal RNFL, temporal and nasal macular choroid, and temporal nerve choroid (right panel) thickness in all groups.

**Figure 3:**
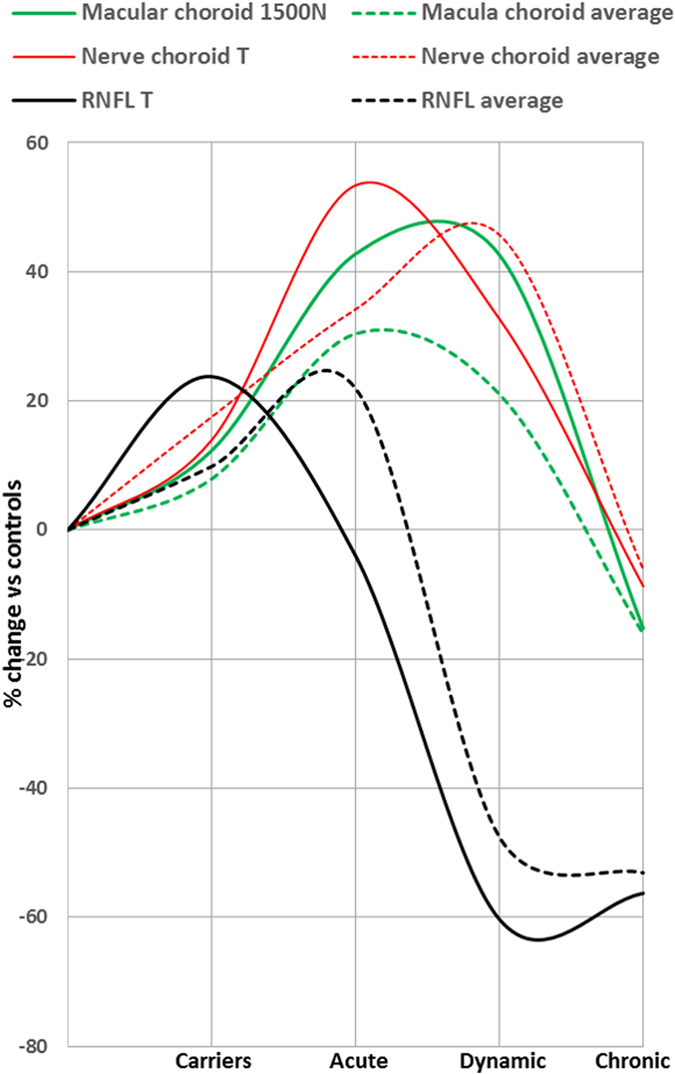
Retinal nerve fiber layer (RNFL) and choroid thickness change in the LHON subjects, stratified into asymptomatic carriers, acute, dynamic and chronic stages. Solid lines indicate average measurements of RNFL, nerve and macular choroid thicknesses, whereas dotted lines indicate measurements corresponding to the papillomacular bundle region, in particular temporal RNFL, nerve choroid temporal and macular choroid nasal thicknesses. Changes are reported as percentage of change from the average value of healthy controls.

Looking at the RNFL and choroid data simultaneously, it appeared that average measurements of both structures progressively increased from healthy subjects to LHON carriers and to A-LHON, followed by a progressive reduction in chronic states (Fig. 2, left panel). However, focusing on the papillomacular region of LHON patients, temporal RNFL thickness tended to decrease already in the acute phase, while peri-optic nerve choroid temporal and macular choroid nasal were clearly thicker (Fig. 2, right panel). Moreover, the choroidal thickening in the acute stage was more evident on temporal peri-optic nerve and nasal macular than in the temporal macular and average measurements [Fig. 2]. A summary of these results is shown in Fig. 3, which emphasizes the timing of choroidal and RNFL thickness changes in the different stages of LHON [Fig. 3].

A sub-analysis of different mutations in both LHON (stratifying the three primary mutations) and DOA (haploinsufficiency vs missense) was also performed, failing to evidence any difference (not shown).

### Correlations

Pearson test showed that LHON disease duration was inversely correlated with the inferior, nasal and temporal macular choroidal thickness measurements. Moreover, we found that average macular choroidal thickness and average RNFL thickness, as well as average peri-optic nerve choroidal thickness and average RNFL thickness, were directly correlated (R^2^ = 0.627 and p < 0.0001, R^2^ = 0.369 and p = 0.013, respectively). Finally, a direct correlation was found between average macular choroidal and average nerve choroidal thicknesses (R^2^ = 0.576 and p < 0.0001).

**Figure 4:**
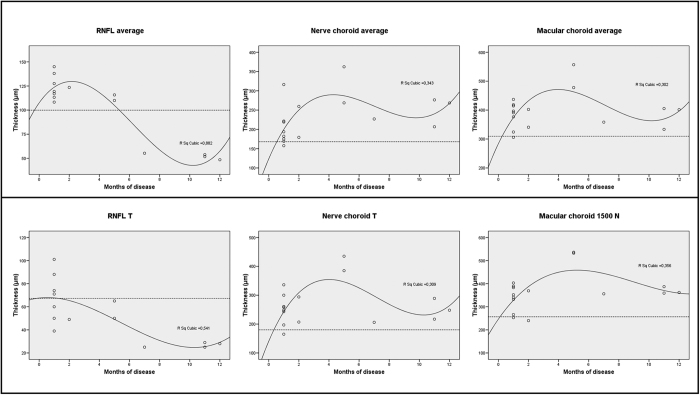
Scatter plots with cubic regression lines, showing the relationship between disease duration in the first 12 months and average measurements of retinal nerve fiber layer (RNFL), nerve and macular choroid thickness (top) and measurements corresponding to the papillomacular bundle region, in particular temporal RNFL, nerve choroid temporal and macular choroid nasal thickness (bottom). Dotted lines indicate mean values of the healthy subjects.

Scatters plots in Fig. 4 show the relationship between disease duration in the first 12 months and average measurements of RNFL, nerve and macular choroid, and measurements corresponding to the papillomacular bundle region, in particular RNFL-T, nerve choroid temporal and macular choroid nasal. The best-fit relationship for all plots was using a cubic regression model, which showed increased thickness, which was followed by thinning until an apparent plateau [Fig. 4]. The plots show that in the acute stage, thickening and subsequent thinning of RNFL start earlier compared with peri-optic nerve and macular choroids. This is more evident in the papillomacular region, where the temporal RNFL thinning is already evident when both temporal nerve and nasal macular choroidal thickening starts [Fig. 4]. After 4–5 months following disease onset, the macular and peri-optic nerve choroidal thinning starts but remains thicker than normal until 12 months, also in the papillomacular region [Fig. 4].

Finally, we failed to find any correlation between choroidal thickness and best corrected visual acuity.

## Discussion

In the present study, we evaluated macular and peripapillary choroidal thickness in LHON patients. Overall, we found that both macular and peripapillary choroidal thicknesses increased in the acute stage. Moreover, we showed a decreased macular choroidal thickness in the chronic stage. Most importantly, we have evidence that the increased thickness of axons preceded the changes of choroidal thickness, suggesting that one event may lead to the other.

It has always been recognized that asymptomatic and acute stages of LHON were characterized by vascular changes, such as peripapillary microangiopathy and vascular telangiectasias^10^,^11^. These signs are more pronounced in the acute phase and, once vision loss stabilizes, the vascular abnormalities fade. Nevertheless, to the best of our knowledge, there is no study that serially evaluated the vascular changes, by means of an *in vivo* imaging approach, across the different LHON stages.

Our group recently reported postmortem evidence of vessel involvement in LHON^26^. Indeed, we demonstrated mitochondrial proliferation affecting both the endothelial and the smooth muscle components of blood vessel walls in LHON patients^26^. Mitochondrial proliferation is part of a thickening of the vessel wall, an increase in the vessel size, as well as a decrease in the lumen diameter, which we have defined as a mitochondrial angiopathy. This finding resembles the hallmark features of mitochondrial encephalomyopathy, lactic acidosis, stroke-like episodes (MELAS) syndrome, and we proposed that in the acute phase of LHON there may be a similar stroke-like episode occurring at the metabolically susceptible optic nerve head.

Taking into account these considerations, the choroidal thickening seen during the LHON acute phase may be part of a compensatory response. Vessels in the choroid may simply be more numerous and or wider, as would happen in response to angiogenic factors (vascular endothelial growth factor–VEGF being just one of the many trophic factors). Such exuberance of vessels is often in consequence to ischemia stimulating hypoxia inducible factor 1-alpha (HIF-1) that in turn causes downstream upregulation of VEGF and other growth factors. HIF-1 is upregulated under conditions of hypoxia^27^, but also through a redox-sensitive mechanism and by reactive oxygen species (ROS)^28^. These conditions of “pseudo-hypoxia” may likely characterize the acute phase of LHON. Finally, increased heat production, secondary to the high metabolic rate and partially uncoupled mitochondrial oxidative phosphorylation due to a complex I defect in LHON, may further aggravate the situation^27^,^28^,^29^. Recently, it has been provided evidence that complex I deficiency may signal a pseudohypoxic state in susceptible tissues such as the RGCs and RNFL^30^. These effects may be additive and not mutually exclusive. Moreover, interestingly, the choroid was found thicker also in carrier patients, but only in the inferior peri-optic nerve sector. The latter aspect could be explained by the fact that the peripapillary inferior quadrant is thinner than the other quadrants in healthy patients, as shown by several studies^31^,^32^, and this thinning could have been missed in patients carrying LHON mutations.

The natural history of peripapillary RNFL changes throughout the different LHON phases has been quantitatively described^8^,^18^,^19^. Our study confirmed a thickening occurring in RNFL layer inferior-temporally, in LHON mutation carriers. The RNFL swelling is likely to depend on the compensatory increase of mitochondrial biogenesis and/or axonal stasis along the fibers. Moreover, we found RNFL temporal thinning in the LHON acute phase, affecting initially the papillomacular bundle. The latter aspect is well described in the acute LHON group, indeed these patients were not tested only at disease onset, but three months later. Finally, the entire RNFL layer was reduced in chronic LHON patients, as well as in DOA subjects.

Despite the cross-sectional nature of our study, the direct correlation between RNFL and choroidal thicknesses at the different disease stages suggested that the changes occurring at these two structures are related. The increase of RFNL thickness preceded the increase of choroid thickness, both in the macula and peripapillary regions, strongly suggesting that one event may drive the other.

We also showed that choroid and RNFL thickness were both reduced in C-LHON and DOA patients. Interestingly, the RNFL was thinner in LHON as compared to DOA, whereas the choroid thickness followed an opposite trend being thicker in LHON than DOA. We propose that in LHON, a low-rate ongoing axonal degeneration as shown at histopathology^1^,^3^,^26^, maintains the signaling for blood vessels to prolong the choroidal changes. We also speculate that in DOA patients, for which RGC and axonal loss occurs over decades, there is a reduction in the overall metabolic rate of the retina accompanied by a corresponding reduction of choroidal thickness. Indeed, there was a direct correlation between RNFL and choroidal thickness.

Limitations of the present study include the cross-sectional nature of the study design. However considering the rarity of LHON, the transition from the asympthomatic to the acute stage is particularly difficult to observe and follow longitudinally. Furthermore, to substantiate the hypothesis of a pseudo-hypoxic tissue condition, promoting the choroidal and vessel changes through the angiogenic signaling, will need specific investigation, both *in vitro* and histopathology level. To this end it might be also instrumental to investigate the now available LHON mouse model^33^. Another limitation is that we did not measure the axial length, which was shown to influence the choroid measurements^34^. However, it should be considered the low variability of refractive error in the enrolled subjects and no patient underwent refractive surgery, thus minimizing the possible influence of axial length on choroidal thickness. Finally, we did not assess test-retest variability in choroidal measurement. Nevertheless, choroidal measurements were shown to have a high intra-observer and interobserver reproducibility^35^.

In conclusion, we provide the first quantitative assessment of choroidal thickness in LHON, as compared to controls and DOA, and we substantiate the involvement of choroidal vessels in correlation to the RNFL changes during the different stages of LHON. The increase in choroidal thickness is a feature of the LHON acute stage, which may follow the pathological events occurring at the RGC and axonal level. Conversely, thinning of the choroidal layer is the common outcome in the chronic stage of LHON and in DOA. Choroid thickness in chronic LHON exceeds that in DOA, possibly as consequence of the still ongoing insidious metabolic neurodegeneration in LHON. Choroidal measures may become a new useful tool to monitor disease activity and efficacy of new therapeutic approaches. We also envisage that the vascular changes occurring in LHON during the acute stage may become a therapeutic target, once their role in LHON pathogenesis is further clarified.

## Additional Information

**How to cite this article**: Borrelli, E. *et al.* Changes in Choroidal Thickness follow the RNFL Changes in Leber’s Hereditary Optic Neuropathy. *Sci. Rep.* **6**, 37332; doi: 10.1038/srep37332 (2016).

**Publisher’s note**: Springer Nature remains neutral with regard to jurisdictional claims in published maps and institutional affiliations.

## Figures and Tables

**Table 1:** Study participants’ demographic and clinical features.

		*Chronic LHON*	*Acute LHON*	*‘Dynamic’ LHON*	*LHON Carriers*	*DOA patients*	*Healthy controls*
*Number of participants (number of eyes analyzed*)		20 (38)	6 (12)	2 (4)	21 (42)	19 (38)	22 (44)
*Gender (number of males*)		16	5	2	10	13	14
*Mutation carried, number of patients (number of eyes analyzed*)							
	***11778*/*ND4***	13 (24)	6 (12)	2 (4)	15 (30)	—	—
	***3460*/*ND1***	5 (10)	0 (0)	0 (0)	4 (8)	—	—
	***14484*/*ND6***	2 (4)	0 (0)	0 (0)	2 (4)	—	—
	***OPA1 H*-*mutation***	—	—	—	—	9 (18)	—
	***OPA1 M*-*mutation***	—	—	—	—	10 (20)	—
*Number of smokers*		2	3		0	2	3
*Age, years*		36.0 ± 10.0	31.5 ± 12.1	36.0 ± 4.2	36.7 ± 13.3	37.5 ± 12.9	39.7 ± 10.1
*Disease duration, years*		11.8 ± 13.8	0.1 ± 0.1	0.7 ± 0.2	—	—	—
*Visual acuity, decimals*		0.11 ± 0.15	0.29 ± 0.43	0.04 ± 0.02	1.00 ± 0.00	0.36 ± 0.23	1.00 ± 0.00

**LHON**: Leber Hereditary Optic Neuropathy; **DOA**: Dominant Optic Atrophy; **H-mutation**: OPA1 haploinsufficiency mutation; **M-mutation**: OPA1 missense mutation.

Data are showed as mean ± SD.

**Table 2:** Comparison of macular and nerve choroidal thickness between LHON patients and the control group.

	Acute LHON patients	Carrier LHON patients	Control group	Acute LHON patients vs Control group	Carrier LHON patients vs Control group	Acute LHON patients vs Carrier LHON patients
Average macular choroidal thickness (μm)	403.4 ± 68.2	334.0 ± 81.6	309.5 ± 38.2	p < 0.0001**	p = 0.255	p = 0.004**
Macular choroidal SF thickness (μm)	425.6 ± 79.3	349.0 ± 90.1	344.1 ± 49.1	p = 0.003**	p = 1.0	p = 0.006**
Macular choroidal 1500 ***mμ*** N thickness (μm)	366.3 ± 95.9	295.3 ± 97.0	256.6 ± 59.2	p < 0.0001**	p = 0.105	p = 0.030*
Macular choroidal 750 ***mμ*** N thickness (μm)	406.9 ± 84.8	325.4 ± 90.7	309.2 ± 57.4	p = 0.001**	p = 1.0	p = 0.005**
Macular choroidal 1500 ***mμ*** S thickness (μm)	413.5 ± 70.4	344.7 ± 89.1	317.6 ± 42.9	p < 0.0001**	p = 0.240	p = 0.014*
Macular choroidal 750 ***mμ*** S thickness (μm)	428.9 ± 62.4	350.8 ± 91.2	318.2 ± 44.2	p < 0.0001**	p = 0.113	p = 0.005**
Macular choroidal 1500 ***mμ*** T thickness (μm)	376.5 ± 59.7	323.7 ± 74.7	300.1 ± 51.3	p = 0.001**	p = 0.284	p = 0.039*
Macular choroidal 750 ***mμ*** T thickness (μm)	398.3 ± 66.4	333.9 ± 81.9	322.3 ± 44.3	p = 0.002**	p = 1.0	p = 0.011*
Macular choroidal 1500 ***mμ*** I thickness (μm)	389.6 ± 85.5	338.5 ± 77.9	303.9 ± 40.3	p = 0.001**	p = 0.051	p = 0.068
Macular choroidal 750 ***mμ*** I thickness (μm)	402.1 ± 73.0	337.9 ± 83.9	313.4 ± 43.7	p = 0.001**	p = 0.303	p = 0.018*
Average nerve choroidal thickness (μm)	225.4 ± 64.2	197.3 ± 52.8	167.9 ± 37.5	p = 0.002**	p = 0.038*	p = 0.263
Nerve choroidal T thickness (μm)	277.4 ± 78.2	206.0 ± 75.4	180.1 ± 37.9	p < 0.0001**	p = 0.286	p = 0.003**
Nerve choroidal S thickness (μm)	244.2 ± 67.1	214.3 ± 59.4	182.3 ± 48.2	p = 0.005**	p = 0.054	p = 0.335
Nerve choroidal N thickness (μm)	206.9 ± 56.1	196.5 ± 50.8	175.2 ± 51.3	p = 0.216	p = 0.253	p = 1.0
Nerve choroidal I thickness (μm)	172.9 ± 80.2	172.6 ± 52.8	133.3 ± 41.0	p = 0.089	p = 0.007**	p = 1.0

**LHON:** Leber Hereditary Optic Neuropathy; **SF**: sub-foveal; **T:** temporal quadrant; **S**: superior quadrant; **N**: nasal quadrant; **I**: inferior quadrant.

Data are showed as mean ± SD. Values were compared by means of one-way analysis of covariance (ANCOVA), followed by Bonferroni post hoc test. Gender was used as covariate in the analysis.

*<0.05, **<0.01.

**Table 3:** Comparison of macular and nerve choroidal thickness among chronic LHON patients, DOA patients and the control group.

	Chronic LHON patients	DOA patients	Control group	*Chronic LHON patients vs Control group*	*DOA patients vs Control group*	*Chronic LHON patients vs DOA patients*
*Average macular choroidal thickness (μm*)	259.8 ± 67.9	250.9 ± 83.4	309.5 ± 38.2	p = 0.002**	p < 0.0001**	p = 1.0
*Macular choroidal SF thickness (μm*)	274.9 ± 77.3	254.4 ± 85.2	344.1 ± 49.1	p < 0.0001**	p < 0.0001**	p = 0.618
*Macular choroidal 1500 **mμ** N thickness (μm*)	217.4 ± 73.0	212.9 ± 95.8	256.6 ± 59.2	p = 0.084	p = 0.032*	p = 1.0
*Macular choroidal 750 **mμ** N thickness (μm*)	252.4 ± 76.8	237.0 ± 85.9	309.2 ± 57.4	p = 0.030*	p < 0.0001**	p = 1.0
*Macular choroidal 1500 **mμ** S thickness (μm*)	260.5 ± 71.3	268.9 ± 79.5	317.6 ± 42.9	p = 0.001**	p = 0.003**	p = 1.0
*Macular choroidal 750 **mμ** S thickness (μm*)	262.8 ± 77.4	261.6 ± 80.81	318.2 ± 44.2	p = 0.002**	p = 0.001**	p = 1.0
*Macular choroidal 1500 **mμ** T thickness (μm*)	257.3 ± 70.5	254.9 ± 80.6	300.1 ± 51.3	p = 0.020*	p = 0.008*	p = 1.0
*Macular choroidal 750 **mμ** T thickness (μm*)	270.4 ± 73.4	257.1 ± 82.6	322.3 ± 44.3	p = 0.030*	p < 0.0001**	p = 1.0
*Macular choroidal 1500 **mμ** I thickness (μm*)	274.3 ± 69.4	260.4 ± 80.5	303.9 ± 40.3	p = 0.143	p = 0.008**	p = 1.0
*Macular choroidal 750 **mμ** I thickness (μm*)	267.9 ± 68.2	255.8 ± 83.4	313.4 ± 43.7	p = 0.001**	p = 0.015*	p = 1.0
*Average nerve choroidal thickness (μm*)	157.4 ± 61.0	144.8 ± 61.0	167.9 ± 37.5	p = 1.0	p = 0.160	p = 0.874
*Nerve choroidal T thickness (μm*)	165.1 ± 70.0	147.6 ± 72.5	180.1 ± 37.9	p = 0.918	p = 0.070	p = 0.689
*Nerve choroidal S thickness (μm*)	172.8 ± 71.0	158.2 ± 66.5	182.3 ± 48.2	p = 1.0	p = 0.291	p = 0.940
*Nerve choroidal N thickness (μm*)	156.5 ± 69.9	150.7 ± 51.7	175.2 ± 51.3	p = 0.521	p = 0.177	p = 1.0
*Nerve choroidal I thickness (μm*)	135.2 ± 56.8	123.0 ± 52.9	133.3 ± 41.0	p = 1.0	p = 1.0	p = 0.900

**LHON:** Leber Hereditary Optic Neuropathy; **DOA:** Dominant Optic Atrophy; **SF**: sub-foveal; **T:** temporal quadrant; **S**: superior quadrant; **N**: nasal quadrant; **I**: inferior quadrant.

Data are showed as mean ± SD. Values were compared between each LHON group and healthy subjects, by means of one-way analysis of covariance (ANCOVA), followed by Bonferroni post hoc test. Gender was used as covariate in the analysis.

*<0.05, **<0.01.

**Table 4:** Comparison of Retinal Nerve Fiber Layer thickness between LHON patients and the control group.

	Acute LHON patients	Carrier LHON patients	Control group	*Acute LHON patients vs Control group*	*Carrier LHON patients vs Control group*	*Acute LHON patients vs Carrier LHON patients*
*RNFL Average thickness (μm*)	121.7 ± 33.1	109.7 ± 10.1	99.9 ± 6.6	p = 0.002**	p = 0.038*	p = 0.001**
*RNFL T thickness (μm*)	64.7 ± 19.3	83.4 ± 15.3	67.38 ± 10.6	p = 1.0	p < 0.0001**	p = 0.001**
*RNFL S thickness (μm*)	169.9 ± 33.4	131.5 ± 14.6	129.3 ± 16.1	p < 0.0001**	p = 1.0	p < 0.0001**
*RNFL N thickness (μm*)	95.2 ± 13.5	82.7 ± 20.1	79.0 ± 16.6	p = 0.061	p = 1.0	p = 0.190
*RNFL I thickness (μm*)	158.1 ± 16.4	144.1 ± 20.5	123.7 ± 10.6	p < 0.0001**	p < 0.0001**	p = 0.018*

**LHON:** Leber Hereditary Optic Neuropathy; **RNFL**: Retinal Nerve Fiber Layer; **SF**: sub-foveal; **T:** temporal quadrant; **S**: superior quadrant; **N**: nasal quadrant; **I**: inferior quadrant.

Data are showed as mean ± SD. Values were compared between each LHON group and healthy subjects, by means of one-way analysis of covariance (ANCOVA), followed by Bonferroni post hoc test. Gender was used as covariate in the analysis.

*<0.05, **<0.01.

**Table 5:** Comparison of Retinal Nerve Fiber Layer thickness among chronic LHON patients, DOA patients and the control group.

	Chronic LHON patients	DOA patients	Control group	*Chronic LHON patients vs Control group*	*DOA patients vs Control group*	*Chronic LHON patients vs DOA patients*
*RNFL Average thickness (μm*)	46.7 ± 11.6	58.6 ± 9.1	99.9 ± 6.6	p < 0.0001**	p < 0.0001**	p < 0.0001**
*RNFL T thickness (μm*)	29.5 ± 15.2	27.6 ± 7.6	67.38 ± 10.6	p < 0.0001**	p < 0.0001**	p = 1.0
*RNFL S thickness (μm*)	62.5 ± 17.2	83.9 ± 13.9	129.3 ± 16.1	p < 0.0001**	p < 0.0001**	p < 0.0001**
*RNFL N thickness (μm*)	38.6 ± 17.9	51.2 ± 11.5	79.0 ± 16.6	p < 0.0001**	p < 0.0001**	p = 0.002**
*RNFL I thickness (μm*)	56.5 ± 11.7	71.8 ± 14.9	123.7 ± 10.6	p < 0.0001**	p < 0.0001**	p < 0.0001**

**LHON:** Leber Hereditary Optic Neuropathy; **DOA:** Dominant Optic Atrophy; **RNFL**: Retinal Nerve Fiber Layer; **SF**: sub-foveal; **T**: temporal quadrant; **S**: superior quadrant; **N**: nasal quadrant; **I**: inferior quadrant.

Data are showed as mean ± SD. Values were compared between each LHON group and healthy subjects, by means of one-way analysis of covariance (ANCOVA), followed by Bonferroni post hoc test. Gender was used as covariate in the analysis.

*<0.05, **<0.01.
